# Open reduction internal fixation of lateral humeral condyle fractures in children. A series of 105 fractures from a single institution

**DOI:** 10.1007/s11751-014-0193-z

**Published:** 2014-07-15

**Authors:** Andreas Leonidou, Krissen Chettiar, Simon Graham, Pouya Akhbari, Konstantinos Antonis, Eleftherios Tsiridis, Omiros Leonidou

**Affiliations:** 1 First Department of Trauma and Orthopaedics, Athens Paediatric Hospital “Agia Sophia”, Thivon and Papadiamantopoulou, Goudi, 11527 Athens, Greece; 2 Division of Surgery, Academic Department of Orthopaedics and Trauma, Aristotle University Medical School, Thessaloníki, Greece

**Keywords:** Lateral condyle fractures, ORIF, Radiological union, Lateral spurring

## Abstract

Lateral humeral condyle fractures account for 17 % of the distal humeral condyle fractures. Displaced and/or rotated fractures require appropriate reduction and stabilisation. There are, however, a number of controversies in the surgical management of these patients. The aim of the present study was to review the results of patients with a displaced lateral humeral condyle fracture treated with open reduction and internal fixation (ORIF). We retrospectively reviewed children treated with ORIF of lateral humeral condyle fractures at a single institution over a period of 13 years. All cases were identified through the trauma register. Case notes and radiographs were retrieved. Fracture classification, mode of fixation, time to union, and final outcomes at the latest follow-up were reviewed. One hundred and five lateral condyle fractures were identified in 76 male and 29 female patients. Average age was 6.2 years. Ninety-two were Milch type II and 13 Milch type I. According to the Jacob’s classification, 38 were type II and 67 type III. All fractures were treated with open reduction and fixation with K-wires. Average time to radiological union was 33 days. Follow-up ranged between 2 and 8 years (average 3.2 years). Radiological hypertrophy of the lateral condyle was present in 45 cases (42 %). Three patients developed a pseudo-cubitus varus deformity. Further four patients developed a true cubitus varus. There was one case of superficial infection of the K-wires and one case of delayed union. At the latest follow-up, 96 % of the patients achieved an excellent final result and 4 % a good final result. Our results demonstrate that fracture union and excellent final outcomes can be expected in all patients using our protocol, whereby all patients with a displaced fracture are managed by ORIF with K-wire fixation, with the wires only being removed after there is evidence of radiological union. Compared to recent reports of closed reduction internal fixation, this series demonstrates good results with no complications directly relating to the open reduction technique. *Level of evidence* Case series, Level IV.

## Introduction

Lateral condyle fractures of the distal humerus are the second most common fractures at the elbow in the paediatric population usually between the ages of 6–10 years old making up 5–20 % of fractures in children [1, 2].

The diagnosis can be difficult both radiologically and clinically, with loss of function occurring, due to extension into the articular surface. The result of an incorrectly treated lateral condylar physeal injury may not be evident until months or years after the initial index injury [3].

The Milch classification is widely used, and they are; type I and type II according to whether the fracture exited through the capitellar–trochlear groove or through the trochlear, respectively [4]. Cotton noted that the fragment was commonly displaced outward and backwards [5]. The Jacob classification dictates whether surgical intervention is required. A Jacob I is non-displaced, II is displaced by 2 mm, but not malrotated. Type III is displacement with malrotation [6]. The aim of lateral humeral condyle fracture treatment is to ensure healing of the fracture and to prevent pseudoarthrosis, malunion, deformities and functional disorders [3]. Traditionally, undisplaced stable fractures were treated in cast immobilisation with observation. Articular fractures that have a hinge may be treated with closed reduction and percutaneous pinning. In certain situations, an arthrogram or an MRI scan may help define articular congruity and adequacy of the reduction [3, 7].

Fractures that are unstable, malrotated and displaced by over 2 mm usually undergo open reduction internal fixation usually with wires, smooth pins or screws [8, 9]. Debate persists as to how much displacement and fracture instability is required before open reduction and internal fixation (ORIF) is indicated [2, 10]. Recently published studies further challenge the necessity of open reduction of a displaced fracture, advocating good results following close reduction and internal fixation (CRIF) of completely displaced and rotated fragments [9].

The aim of the present study was to review the results of patients with a displaced lateral humeral condyle fracture treated with ORIF over a 13-year period at a paediatric tertiary referral centre.

## Methods

A retrospective study of all patients presenting with a displaced paediatric lateral humeral condyle fracture to our tertiary Paediatric Orthopaedic Unit between 1993 and 2011 was conducted. Approval to perform our study was obtained by the Institutional Ethics Committee for Human Research.

Initial assessment of the patients was performed in the Accident and Emergency Department of our Institution. The injured limb was examined for deformity, wounds and neurovascular integrity. Antero–posterior, oblique and lateral radiographs of the elbow were routinely performed. Fractures were classified using the Milch as well as the Jacob classification. The acceptable displacement for conservative management in an above elbow plaster of Paris (POP) cast was up to 2 mm. Patients who were treated conservatively were closely followed up with radiographs every week to ensure that the fracture has not displaced. The POP cast was removed upon radiological union—typically between 4 and 6 weeks—and physiotherapy commenced.

Following anaesthetic assessment, all patients with a displaced lateral humeral condyle fracture were consented and listed for ORIF with Kirschner wires (K-wires) in the operating theatre. All fractures were treated by a Consultant Paediatric Orthopaedic Surgeon as earlier as starvation status and emergency theatres facility allowed access. A single dose of intravenous prophylactic antibiotics was administered at the anaesthetic induction, as per hospital policy, and tourniquet was used. The fracture was identified and reduced via a dorsolateral approach to the distal humerus, through the interval between brachioradialis and triceps. The joint surface was accurately reduced with minimal dissection of soft tissues from the distal fragment in order to reduce the risk of avascular necrosis of the capitellum. The reduction was stabilised with two divergent K-wires that were left outside the skin. Subsequently, an above elbow POP in neutral position was applied.

**Fig. 1:**
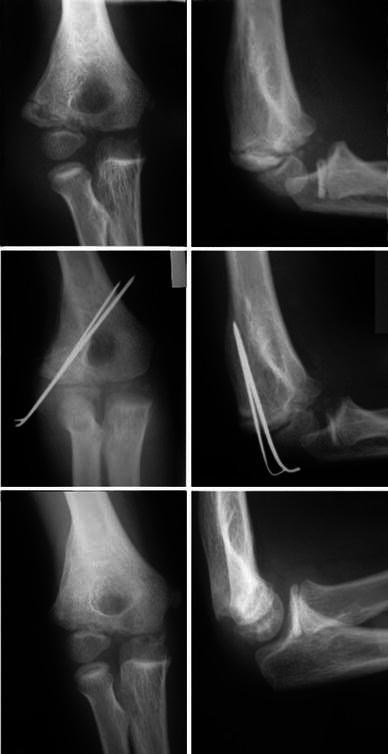
9-year-old male, fracture healed at 5 weeks, X-ray at 1 year demonstrates healing with lateral hypertrophy

Patients were followed up weekly until radiological union of the fracture was evident (Fig. 1), and thereafter, the wires and the POP were removed in the outpatient department without the use of general or local anaesthetic. Following the removal of plaster, all patients were mobilised with intensive physiotherapy focusing on elbow full range of movement (ROM), mainly with active movement exercises.

According to the institutional protocols, our patients were followed up until skeletal maturity to assess residual or late deformities. At the final follow-up appointment before discharge, the patient’s outcome was assessed clinically for ROM and deformity and radiologically. Also, the patients were asked about any residual pain and whether or not they were happy performing daily life activities and sports. The results were graded according to the criteria suggested by Hardacre et al. (Table 1) [11].

**Table 1:** Assessing the results of treatment in patients with lateral humeral condyle fractures as described by Hardacre et al. [10]

Excellent Result	No symptoms + Full ROM + No alteration in the carrying angle
Good Result	ROM deficit < 15o of complete extension + minimal alteration in the carrying angle + pain apart from arthritic/neurological pain
Poor Result	Disabling loss of motion, conspicuous alteration of carrying angle, arthritic symptoms, ulnar neuritis, non-union and avascular necrosis.

## Results

One hundred and five patients with a displaced paediatric lateral humeral condyle fracture were identified and included in the study, 76 males and 29 females. The age of the patients ranged between 3 and 13 years, with a mean of 6.2 years. All included cases were the result of low-energy closed injuries. In relation to the Milch’s classification, 13 fractures were classified as Milch I and 92 as Milch II. According to the Jacobs classification for displacement, 38 fractures were classified as type II and 67 as type III.

The mean time to radiological union of the fracture and therefore removal of the wires was 33 days (4.7 weeks). Radiological union ranged between 21 and 56 days. (3–8 weeks). All the K-wires were removed in the outpatient department.

One patient had a superficial infection around the K-wires, which responded well and eventually resolved with the administration of oral antibiotics. The majority of the fractures demonstrated radiological union between 4 and 6 weeks with the exception of one patient with a Jacob III fracture who reached 8 weeks.

Follow-up ranged between 2 and 8 years with an average of 3.2 years. At the final appointment, all patients had achieved full range of movement of the elbow joint. Furthermore, there were no cases of residual pain, and all patients were happy performing daily life activities and participating in sports. None of the patients in this series developed a non-union or a malunion.

**Fig. 2:**
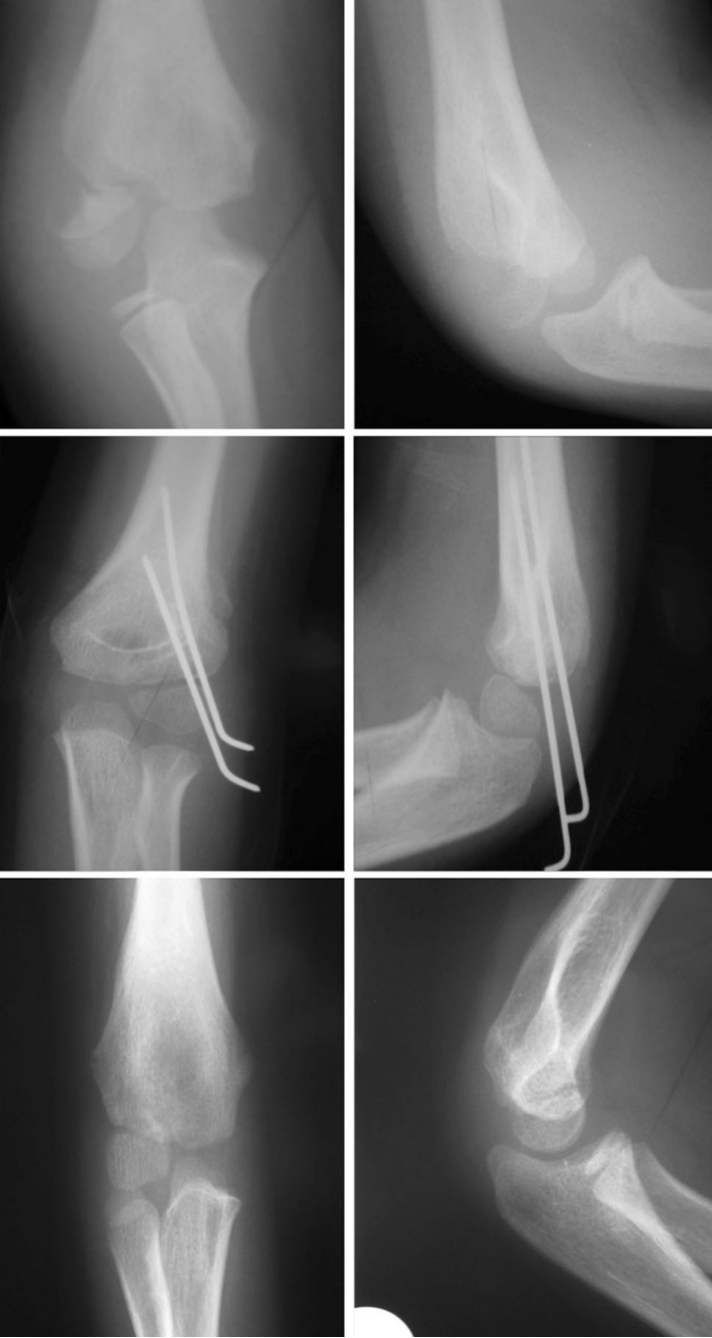
6-year-old male, fracture healed at 5 weeks, X-ray at 1 year demonstrates healing without lateral hypertrophy

Following our management lateral spurring (hypertrophy of the lateral condyle) occurred in 45 cases (42 %). As a result of lateral spurring, 3 patients developed a pseudo-cubitus varus deformity. Further, 4 patients developed a true cubitus varus of less than 5^o^. In all cases of lateral spurring and cubitus varus, there was no pain or interference with daily activities, and sports and no corrective intervention was required. None of the patients developed a fishtail deformity. Figures 2 and 1 demonstrate, respectively, a case where the fracture healed without radial hypertrophy and a case that lateral spurring occurred. According to the criteria by Hardacre et al, 101 patients (96 %) achieved an excellent final result, 4 patients (4 %) achieved good final results, and no patient achieved a poor result.

## Discussion

The results of our study demonstrate that open reduction and K-wire fixation of displaced (>2 mm) lateral humeral condyle fractures leads to excellent clinical and radiological results without any significant complications. The necessity of reduction and stabilisation of displaced and/or rotated lateral condyle fractures has been well established in the literature [3, 10, 12]. There are, however, a number of controversies in the surgical management of these patients.

The first controversy is as to whether displaced and rotated lateral condyle fractures should be managed with ORIF or with CRIF [10]. Advocates of close reduction hypothesise that ORIF might be unnecessary in many cases and that it might even lead to avascular necrosis as a result of extensive soft tissue dissection [10]. Song et al. [9] prospectively looked at 63 patients with lateral condyle fractures of the humerus. They attempted closed reduction internal fixation using K-wires in all of them, but in 13 cases ORIF was required. Their success rate for fixation was 73 % with no cases of non-union or malunion. They suggested that CRIF often results in effective treatment for displaced lateral condyle fractures. However, in their study, only 3 of the 6 patients with a Jacob III fracture were managed with closed reduction [9]. In a subsequent study, Song et al. [13] prospectively looked at 24 Jacob III lateral condyle fractures. Of these, 18 were managed with CRIF and 6 with ORIF using K-wires. It is of note that out of the 6 cases of ORIF, 3 were the results of the surgeon’s lack of confidence and experience, according to the authors [13]. The vast majority of the cases in both studies by Song et al. [9, 13] were managed by one experienced paediatric orthopaedic surgeon, suggesting that close reduction could work better in the hands of more experienced surgeons. In our study, we presented 67 cases of Jacob III fractures treated successfully with ORIF with only one case of delayed union and no cases of avascular necrosis. We therefore advocate for ORIF in all displaced lateral condyle fracture as our results demonstrated union and good functional outcome in all patients with no significant complications.

A second controversy exists as to when the K-wires should be removed. Thomas et al. [12] managed 104 cases of displaced lateral condyle fractures of the humerus with ORIF and K-wire fixation. They advocated that 3 weeks of K-wire stabilisation is sufficient for the fracture to heal and therefore removed all the wires and began elbow mobilisation after the elapse of this period [12]. The authors reported only one case of delayed union in a patient whose K-wires were removed at 19 days [12]. In the present study, we removed the K-wire only after radiological union was evident. Even though we had cases with union and subsequent removal of wires at 3 weeks, the mean time to radiological union of the fracture was 33 days (4.7 weeks). Consequently, we advise for the K-wires to be removed once radiological union is evident. In agreement to our suggestion is the recent paper by Song and Waters [10]. The authors mentioned that displaced fractures should be stabilised until they are healed radiographically [10].

In our study, all the K-wires were left exposed. It has been stipulated that leaving the wires exposed could increase the risk of infection with reported incidences varying from 1 to 28 % [8]. The authors of the present study believe that leaving the wires exposed carries the advantage of wire removal in the outpatient department instead of administrating a further general anaesthetic to the patient. Furthermore, from our series, only 1 case out of 105 (0.9 %) developed a superficial infection around the K-wires, which was successfully treated with oral antibiotics. There were no cases of deep infection. In agreement to our practice is the study by Das De et al. [8], which advocates for leaving the wires exposed following ORIF of a lateral condyle fracture. In our study, all patients received a single dose of prophylactic antibiotics at induction. It has been debated whether prophylactic antibiotics should be used for percutaneous wiring of fractures and some authors advise against it [14, 15]. Nevertheless, in our cohort, all patients underwent open reduction and subsequent wire fixation, and therefore, the authors felt that prophylactic antibiotics should be used, which is in accordance with our institutional policy. Several authors are in agreement that prophylactic antibiotics should be used for open orthopaedic procedures where a foreign material is inserted [16, 17].

Bony overgrowth (lateral spurring) over the lateral condyle is a distinct radiological finding commonly seen in children following a fracture of the lateral condyle of the humerus [12, 18]. A recent study by Pribaz et al. [19] consisting of 212 lateral condyle fractures treated by various methods, demonstrated that 73 % of the patients developed some degree of lateral spur. The development and size of the spur was positively correlated with the degree of initial fracture displacement [19]. Furthermore, they noted that lateral spurring was more common in patients treated surgically (incidence 91 %) compared with those managed conservatively (incidence 59 %). However, they did not find a significant difference between those managed with CRIF compared with ORIF [19]. The authors concluded that the increased incidence of spurring in the surgically treated group is related to the increased fracture displacement at the time of the injury [19]. In our series, 44 cases (42 %) of the patients developed lateral spurring. Similar incidence of lateral spurring (40 %) was reported in the study by Thomas et al. [12]. As a sequela of lateral spurring, 3 of our patients developed a pseudo-cubitus varus deformity at the elbow. Although the patients were able to feel the spur, it was pain free and did not affect their range of movements nor interfered with their daily activities and sports, which is in accordance to the published literature [12, 19].

Cubitus varus angulation has been a documented complication of lateral condyle fractures [20–22]. The incidence of cubitus varus is not positively related to either surgical or conservative management [20]. The deformity is most of the times benign and very rarely causes symptoms and requires surgical correction [20–22]. In our study, four patients (4 %) developed a true cubitus varus of less than 5^o^. This was mainly a cosmetic deformity not affecting patients’ quality of life, and therefore, no corrective osteotomies were required.

Growth disturbance can occur after a lateral humeral condyle fracture in the form of a partial lateral growth plate closure or partial closure of the centre of the physis. In the latter case, a persistent gap between the lateral condylar physis and the trochlea could lead to a sharp angle wedge deformity also known as “fishtail deformity” [3]. Fishtail deformity can lead to cubitus varus and usually does not cause any functional problems or requires surgical intervention [3]. Several authors have correlated fishtail deformity with inadequately reduced fractures [23, 24]. In our cohort, all the patients were treated with open reduction, and none of them subsequently developed a fishtail deformity. This further emphasises the importance of achieving accurate anatomic reduction in these patients.

Treatment of lateral humeral condyle fractures should ensure that patients are not exposed to unnecessary radiation. During the operation, screening must be kept to a minimum and the patient should be covered with a lead radioprotective apron. During follow-up, only one radiograph per week until bone healing is established and then 6 months—yearly radiographs until skeletal maturity when indicated. Different authors report low effective doses of radiation following an elbow radiograph between 0.01 and 0.05 mSV, which is the equivalent period of natural background radiation of a few days and carries no increased risk for severe complications and cancer development [25, 26].

In 1971, Hardacre et al. [11] presented their criteria for grading the outcomes following treatment of lateral humeral condyle fractures, taking into consideration symptoms, range of motion and deformity. These criteria have been used in several other studies for assessing outcomes in lateral condyle fractures [2, 9, 13]. In the author’s opinion, this grading system as used in the present study was easy to utilise and corresponded well with the patient’s clinical outcome.

To the best of our knowledge, this study presents the third larger single-centre series of surgical management of paediatric lateral condyle fractures recorded in the English language over the last 20 years. The good follow-up of our patients provides useful information on the outcome of the surgical management of displaced lateral condyle fractures. The main limitation of the study was its retrospective nature.

Our results demonstrate that fracture union and excellent final outcomes can be expected in all patients using our protocol, whereby all patients with a displaced fracture are managed by ORIF with K-wire fixation, with the wires only being removed after there is evidence of radiological union. Physiotherapy as soon as possible after the immobilisation period is important as it has been shown to be related with fewer complications, fewer residual symptoms and faster gains in range of motion and strength [27]. The authors believe that the Jacob classification system is sufficient to guide treatment with focus on the necessity to reduce fractures displaced more than 2 mm. On the basis of the good outcomes and no significant complications in cases of Jacob III fractures, we advocate for open reduction of these injuries as opposed to the proposed closed reduction by some studies. Furthermore, our results confirm that lateral spurring—even though frequent—does not cause any symptoms. Our study adds evidence on the outcomes of surgically treated lateral humeral condyle fractures and contributes to the clarification of the associated controversies.
